# Vaccination Coverage by Age 24 Months Among Children Born in 2015 and 2016 — National Immunization Survey-Child, United States, 2016–2018

**DOI:** 10.15585/mmwr.mm6841e2

**Published:** 2019-10-18

**Authors:** Holly A. Hill, James A. Singleton, David Yankey, Laurie D. Elam-Evans, S. Cassandra Pingali, Yoonjae Kang

**Affiliations:** 1 Immunization Services Division, National Center for Immunization and Respiratory Diseases, CDC.

The Advisory Committee on Immunization Practices (ACIP) recommends that children be vaccinated against 14 potentially serious illnesses during the first 24 months of life (*1*). CDC used data from the National Immunization Survey-Child (NIS-Child) to assess vaccination coverage with the recommended number of doses of each vaccine at the national, state, territorial, and selected local levels* among children born in 2015 and 2016. Coverage by age 24 months was at least 90% nationally for ≥3 doses of poliovirus vaccine, ≥1 dose of measles, mumps, and rubella vaccine (MMR), ≥3 doses of hepatitis B vaccine (HepB), and ≥1 dose of varicella vaccine, although MMR coverage was <90% in 20 states. Children were least likely to be up to date by age 24 months with ≥2 doses of influenza vaccine (56.6%). Only 1.3% of children born in 2015 and 2016 had received no vaccinations by the second birthday. Coverage was lower for uninsured children and for children insured by Medicaid than for those with private health insurance. Vaccination coverage can be increased by improving access to vaccine providers and eliminating missed opportunities to vaccinate children during health care visits. Increased use of local vaccination coverage data is needed to identify communities at higher risk for outbreaks of measles and other vaccine-preventable diseases.

The NIS-Child is a random-digit–dialed telephone survey^†^ of parents or guardians of children aged 19–35 months. Respondents are asked to provide contact information for all providers who administered vaccines to their children. With parental consent, a survey is mailed to each identified provider, requesting the child’s vaccination history. Multiple responses for an individual child are synthesized into a comprehensive vaccination history which is used to estimate vaccination coverage. To estimate coverage for the 25,059 children with adequate provider data^§^ born in 2015 and 2016, NIS-Child data from 2016–2018 were combined; for survey year 2018, the Council of American Survey Research Organizations’ response rate was 24.6%, and 54.0% of children with household interviews had adequate provider data.^¶^ With this report, CDC has transitioned to reporting NIS-Child data by birth year rather than survey year. Vaccination coverage by age 24 months was estimated using Kaplan-Meier (time to event) analysis to account for children who were aged <24 months on the date vaccination status was assessed. Coverage with ≥2 doses of hepatitis A vaccine (HepA) was assessed at 35 months (the maximum age included in the survey), because the second dose of HepA can be administered as late as age 41 months under the current schedule. Previous NIS-Child weighting methods were modified to optimize estimation by birth year and to reflect the shift from a dual landline and cellular telephone sample frame to an exclusively cellular telephone sampling frame in 2018.** Differences in coverage estimates were evaluated using t-tests on weighted data; p-values of <0.05 were considered statistically significant. Analyses were performed using SAS (version 9.4; SAS institute) and SUDAAN (version 11.0.1; Research Triangle Institute). No evidence for a change in survey accuracy from the 2017 to 2018 survey year was detected (https://www.cdc.gov/vaccines/imz-managers/coverage/childvaxview/pubs-presentations/NIS-child-vac-coverage-estimates-2014-2018-tables.html#supp-table-01) (*2*).

## National Vaccination Coverage

Coverage by age 24 months was ≥90% for ≥3 doses of poliovirus vaccine (92.7%), ≥1 dose of MMR (90.4%), ≥3 doses of HepB (91.0%), and ≥1 dose of varicella vaccine (90.0%) (Table 1). Compared with estimates for children born in 2013 and 2014, coverage for children born during 2015–2016 increased for the HepB birth dose (3.2 percentage points), ≥1 dose of HepA (1.5 percentage points), and ≥2 doses of influenza vaccine (3.6 percentage points). Coverage with ≥2 HepA doses by age 35 months increased from 74.0% for children born during 2013–2014 to 76.6% for children born during 2015–2016. Children were least likely to be up to date by age 24 months with ≥2 doses of influenza vaccine (56.6%) and the combined 7-vaccine series^††^ (68.5%).

**TABLE 1:** Estimated vaccination coverage by age 24 months* among children born during 2013–2016 for selected vaccines and doses — National Immunization Survey-Child, United States, 2014–2018

Vaccine/Dose	% (95% CI)		
	Birth years^†^		Difference (2013–2014) to (2015–2016)
	2013–2014	2015–2016	
**DTaP^§^**			
≥3 doses	93.6 (93.0 to 94.2)	93.8 (93.1 to 94.5)	0.2 (−0.7 to 1.1)
≥4 doses	80.6 (79.7 to 81.6)	80.3 (79.0 to 81.5)	−0.4 (−1.9 to 1.2)
**Poliovirus (≥3 doses)**	91.7 (91.0 to 92.4)	92.7 (92.0 to 93.4)	1.0 (0.0 to 2.0)
**MMR (≥1 dose)^¶^**	90.0 (89.3 to 90.7)	90.4 (89.5 to 91.2)	0.3 (−0.8 to 1.5)
Hib**			
Primary series	92.7 (92.1 to 93.3)	92.7 (91.8 to 93.5)	0.0 (−1.1 to 1.0)
Full series	80.2 (79.3 to 81.1)	79.6 (78.3 to 80.9)	−0.6 (−2.1 to 1.0)
**HepB**			
Birth dose^††^	71.8 (70.7 to 72.8)	75.0 (73.7 to 76.2)	3.2 (1.6 to 4.9)^§§^
≥3 doses	90.9 (90.2 to 91.6)	91.0 (90.2 to 91.9)	0.1 (−1.0 to 1.2)
**Varicella (≥1 dose)^¶^**	89.3 (88.6 to 90.1)	90.0 (89.1 to 90.9)	0.7 (−0.5 to 1.8)
**PCV**			
≥3 doses	91.9 (91.2 to 92.5)	92.0 (91.1 to 92.8)	0.1 (−1.0 to 1.2)
≥4 doses	81.5 (80.6 to 82.4)	81.0 (79.8 to 82.3)	−0.4 (−2.0 to 1.1)
**HepA**			
≥1 dose	83.2 (82.4 to 84.1)	84.7 (83.6 to 85.8)	1.5 (0.1 to 2.9)^§§^
≥2 doses (by 35 months)	74.0 (72.8 to 75.3)	76.6 (74.7 to 78.4)	2.6 (0.4 to 4.8)^§§^
**Rotavirus (by 8 months)^¶¶^**	72.4 (71.3 to 73.4)	73.6 (72.2 to 74.9)	1.2 (−0.5 to 2.9)
Influenza (≥2 doses)***	53.0 (51.9 to 54.1)	56.6 (55.2 to 58.0)	3.6 (1.8 to 5.4)^§§^
**Combined 7-vaccine series^†††^**	68.4 (67.3 to 69.5)	68.5 (67.1 to 69.9)	0.1 (−1.7 to 1.9)
**No vaccinations**	1.1 (1.0 to 1.3)	1.3 (1.1 to 1.5)	0.1 (−0.2 to 0.4)

**Abbreviations:** CI = confidence interval; DTaP = diphtheria, tetanus toxoids, and acellular pertussis vaccine; HepA = hepatitis A vaccine; HepB = hepatitis B vaccine; Hib = *Haemophilus influenzae* type b conjugate vaccine; MMR = measles, mumps, and rubella vaccine; PCV = pneumococcal conjugate vaccine.

* Includes vaccinations received by age 24 months (before the day the child turns 24 months), except for the HepB birth dose, rotavirus vaccination, and ≥2 HepA doses by 35 months. For all vaccines, except the HepB birth dose and rotavirus vaccination, the Kaplan-Meier method was used to estimate vaccination coverage to account for children whose vaccination history was ascertained before age 24 months (35 months for ≥2 HepA doses).

^†^ Data for the 2013 birth year are from survey years 2014, 2015, and 2016; data for the 2014 birth year are from survey years 2015, 2016, and 2017; data for the 2015 birth year are from survey years 2016, 2017, and 2018; data for the 2016 birth year are considered preliminary and come from survey years 2017 and 2018 (data from survey year 2019 are not yet available).

^§^ Includes children who might have received diphtheria and tetanus toxoids vaccine or diphtheria, tetanus toxoids, and pertussis vaccine.

^¶^ Includes children who might have received measles, mumps, rubella, and varicella combination vaccine.

** Hib primary series: receipt of ≥2 or ≥3 doses, depending on product type received; full series: primary series and booster dose, which includes receipt of ≥3 or ≥4 doses, depending on product type received.

^††^ One dose HepB administered from birth through age 3 days.

^§§^ Statistically significantly different from 0 at p<0.05.

^¶¶^ Includes ≥2 doses of Rotarix monovalent rotavirus vaccine, or ≥3 doses of RotaTeq pentavalent rotavirus vaccine. The maximum age for the final rotavirus dose is 8 months, 0 days.

*** Doses must be at least 24 days apart (4 weeks with a 4-day grace period).

^†††^ The combined 7-vaccine series (4:3:1:3*:3:1:4) includes ≥4 doses of DTaP, ≥3 doses of poliovirus vaccine, ≥1 dose of measles-containing vaccine, the full series of Hib (≥3 or ≥4 doses, depending on product type), ≥3 doses of HepB, ≥1 dose of varicella vaccine, and ≥4 doses of PCV.

## Vaccination Coverage by Selected Characteristics and Geographic Location

For most of the vaccines assessed, uninsured children, and children with Medicaid or other nonprivate insurance, had lower coverage than did privately insured children (Table 2). Compared with privately insured children, coverage disparities were largest among uninsured children, ranging from 7.8 percentage points for the HepB birth dose to 33.8 percentage points for ≥2 doses of influenza vaccine. The proportion of children who received no vaccinations was higher among uninsured children (7.4%) than among those with private insurance (0.8%). Disparities were also observed for race/ethnicity (Supplementary Table 1, https://stacks.cdc.gov/view/cdc/81681), poverty level (Supplementary Table 2, https://stacks.cdc.gov/view/cdc/81682), and metropolitan statistical area^§§^ (MSA) (Supplementary Table 2, https://stacks.cdc.gov/view/cdc/81682) but tended to be smaller than those seen with health insurance status. Coverage varied widely by state/local area for many vaccines (Supplementary Table 3, https://stacks.cdc.gov/view/cdc/81683). Coverage with ≥1 dose of MMR was <90% in 20 states; only six states had coverage of 94% or higher (Figure).

**TABLE 2:** Estimated vaccination coverage by age 24 months* among children born during 2015–2016,^†^ by selected vaccines and doses and health insurance status^§^ — National Immunization Survey-Child, United States, 2016–2018

Vaccine/Dose	Health insurance status, % (95% CI)			
	Private only (referent) (n = 12,702)	Any Medicaid (n = 9,442)	Other insurance (n = 2,141)	Uninsured (n = 774)
**DTaP^¶^**				
≥3 doses	96.9 (96.3–97.5)	91.8 (90.5–93.1)**	93.9 (92.2–95.3)**	80.6 (75.2–85.5)**
≥4 doses	87.1 (85.7–88.5)	75.8 (73.6–77.9)**	78.8 (75.4–82.0)**	59.8 (53.8–65.9)**
**Poliovirus (≥3 doses)**	96.1 (95.4–96.7)	90.7 (89.3–92.0)**	92.3 (90.4–94.0)**	79.3 (73.9–84.3)**
**MMR (≥1 dose)^††^**	93.7 (92.8–94.5)	88.6 (87.0–90.1)**	89.8 (87.6–91.8)**	73.2 (67.4–78.7)**
**Hib^§§^**				
Primary series	95.7 (94.5–96.8)	90.7 (89.3–92.1)**	93.7 (91.9–95.1)	78.4 (72.8–83.5)**
Full series	85.5 (83.7–87.1)	75.9 (73.8–78.0)**	79.1 (75.8–82.1)**	58.1 (52.1–64.2)**
**HepB**				
Birth dose^¶¶^	75.6 (73.9–77.2)	76.1 (74.0–78.1)	68.2 (64.3–71.9)**	67.8 (61.9–73.2)**
≥3 doses	93.0 (91.8–94.0)	90.0 (88.5–91.4)**	91.9 (89.9–93.6)	78.6 (73.3–83.5)**
**Varicella (≥1 dose)^††^**	93.2 (92.3–94.0)	88.6 (86.9–90.1)**	89.1 (86.8–91.2)**	70.3 (64.5–75.9)**
**PCV**				
≥3 doses	94.9 (93.5–96.0)	90.3 (88.9–91.7)**	92.0 (90.1–93.7)**	77.2 (71.7–82.4)**
≥4 doses	87.3 (85.6–88.8)	76.8 (74.7–78.9)**	80.9 (77.7–83.9)**	62.5 (56.7–68.3)**
**HepA**				
≥1 dose	87.5 (85.9–89.0)	83.7 (81.9–85.4)**	84.0 (81.2–86.6)**	65.5 (59.7–71.3)**
≥2 doses (by 35 months)	80.5 (77.9–83.1)	75.2 (72.2–78.0)**	76.8 (71.3–81.9)	48.2 (41.0–56.0)**
Rotavirus (by 8 months)***	83.5 (81.9–85.0)	65.9 (63.5–68.1)**	72.4 (68.5–76.0)**	59.8 (53.8–65.5)**
**Influenza (≥2 doses)^†††^**	68.5 (66.6–70.4)	48.2 (45.9–50.5)**	52.7 (48.6–56.9)**	34.7 (29.4–40.7)**
**Combined 7-vaccine series^§§§^**	75.4 (73.5–77.2)	64.3 (62.0–66.6)**	65.9 (62.1–69.6)**	46.7 (40.9–52.9)**
**No vaccinations**	0.8 (0.6–1.0)	1.2 (0.9–1.6)	1.8 (1.2–2.6)**	7.4 (4.7–10.7)**

**Abbreviations:** CI = confidence interval; DTaP = diphtheria, tetanus toxoids, and acellular pertussis vaccine; HepA = hepatitis A vaccine; HepB = hepatitis B vaccine; Hib = *Haemophilus influenzae* type b conjugate vaccine; MMR = measles, mumps, and rubella vaccine; PCV = pneumococcal conjugate vaccine.

* Includes vaccinations received by age 24 months (before the day the child turns 24 months), except for the HepB birth dose, rotavirus vaccination, and ≥2 HepA doses by 35 months. For all vaccines, except the HepB birth dose and rotavirus vaccination, the Kaplan-Meier method was used to estimate vaccination coverage to account for children whose vaccination history was ascertained before age 24 months (35 months for ≥2 HepA doses).

^†^ Data for the 2015 birth year are from survey years 2016, 2017, and 2018; data for the 2016 birth year are considered preliminary and come from survey years 2017 and 2018 (data from survey year 2019 are not yet available).

^§^ Children’s health insurance status was reported by parent or guardian. “Other insurance” includes the Children’s Health Insurance Program, military insurance, coverage via the Indian Health Service, and any other type of health insurance not mentioned elsewhere.

^¶^ Includes children who might have received diphtheria and tetanus toxoids vaccine or diphtheria, tetanus toxoids, and pertussis vaccine.

** Statistically significant (p<0.05) difference compared with the referent group.

^††^ Includes children who might have received measles, mumps, rubella, and varicella combination vaccine.

^§§^ Hib primary series: receipt of ≥2 or ≥3 doses, depending on product type received; full series: primary series and booster dose, which includes receipt of ≥3 or ≥4 doses, depending on product type received.

^¶¶^ One dose HepB administered from birth through age 3 days.

*** Includes ≥2 doses of Rotarix monovalent rotavirus vaccine (RV1), or ≥3 doses of RotaTeq pentavalent rotavirus vaccine (RV5). The maximum age for the final rotavirus dose is 8 months, 0 days.

^†††^ Doses must be at least 24 days apart (4 weeks with a 4-day grace period).

^§§§^ The combined 7-vaccine series (4:3:1:3*:3:1:4) includes ≥4 doses of DTaP, ≥3 doses of poliovirus vaccine, ≥1 dose of measles-containing vaccine, the full series of Hib (≥3 or ≥4 doses, depending on product type), ≥3 doses of HepB, ≥1 dose of varicella vaccine, and ≥4 doses of PCV.

**FIGURE:**
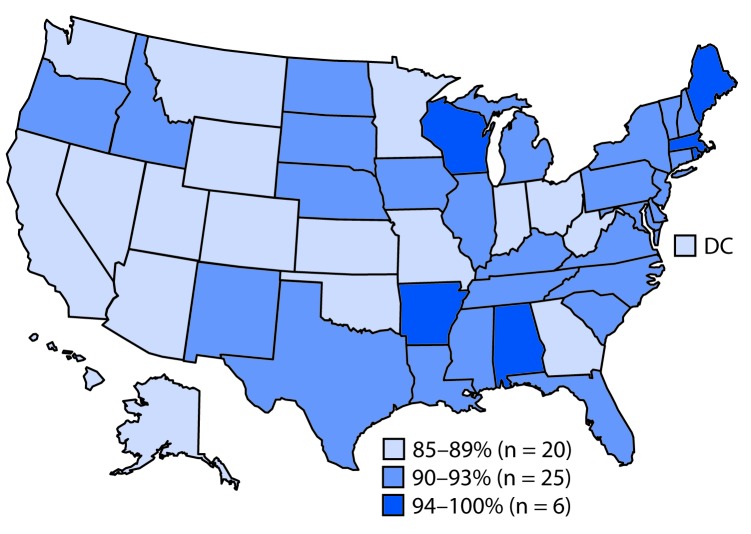
Estimated coverage with ≥1 dose of MMR by age 24 months among children born 2015–2016* — National Immunization Survey-Child, United States, 2016–2018 **Abbreviations:** DC = District of Columbia; MMR = measles, mumps, and rubella vaccine. * Data for the 2015 birth year are from survey years 2016, 2017, and 2018; data for the 2016 birth year are considered preliminary and come from survey years 2017 and 2018 (data from survey year 2019 are not yet available).

## Trends in Vaccination Coverage

Vaccination coverage was stable by single birth year from 2011 through 2016 (https://www.cdc.gov/vaccines/imz-managers/coverage/childvaxview/pubs-presentations/NIS-child-vac-coverage-estimates-2014-2018-tables.html#supp-figure-01), except for an increase in ≥2 doses of HepA by age 35 months from 71.1% (2011) to 76.6% (2016). The proportion of children that received no vaccinations by age 24 months increased slightly across birth years 2011 through 2016, with an estimated change per year of 0.09 percentage points (https://www.cdc.gov/vaccines/imz-managers/coverage/childvaxview/pubs-presentations/NIS-child-vac-coverage-estimates-2014-2018-tables.html#supp-figure-02). Only 1.3% of children born in 2015 and 2016 received no vaccinations (Table 1).

## Discussion

Vaccination coverage by the second birthday among children born during 2015–2016 remained high, with small increases in coverage with hepatitis A and B and influenza vaccines; only 1.3% of children received no vaccinations. However, several opportunities for improvement were apparent. Coverage was lower for children without private health insurance, especially those with no insurance, as well as those living below the poverty level and in more rural areas. Vaccination coverage also varied by state, with 20 states having MMR coverage <90%. Coverage with ≥2 doses of influenza vaccine was the lowest among all recommended childhood vaccines.

The importance of achieving and sustaining high vaccination coverage across all communities is illustrated by the 22 measles outbreaks occurring in the United States in 2019, with 1,249 measles cases identified during January 1–October 1, 2019 (*3*). Most cases have been among persons who were not vaccinated against measles. Pockets of low vaccination coverage, because of lack of access to vaccination services or to hesitancy resulting from the spread of inaccurate information about vaccines, increase the likelihood of a measles outbreak. Strategies are needed to increase access to vaccination services, identify communities at risk, and implement initiatives to counter inaccurate vaccine information (*4*).

Lower vaccination coverage among children who are uninsured, insured by Medicaid or other nonprivate insurance, living below the poverty level, and living in rural areas suggests challenges with access to affordable vaccinations or optimal vaccination services. Uninsured children are eligible for vaccine at no cost through the Vaccines for Children^¶¶^ program, but efforts to promote the program might not be reaching this population and therefore might need to be modified. Targeted programs to address logistical issues such as expanded office hours and transportation to vaccination appointments could facilitate access to vaccination services, regardless of the child’s type of insurance. Providers need to use every patient encounter to screen for and offer vaccinations. An analysis of NIS-Child data for children born during 2005–2015 found that disparities in coverage with ≥4 doses of diphtheria, tetanus toxoids, and acellular pertussis vaccine (DTaP) for those with Medicaid compared with those with private health insurance could have been reduced by 42% had opportunities for receipt of the fourth DTaP dose not been missed during visits when other vaccinations were received (*5*).

The transition to reporting by birth year rather than by survey year more directly assesses recent changes in vaccination coverage and provides more interpretable estimates and more accurate comparisons to evaluate immunization information systems (*2*,*6*,*7*). With a standard age at assessment (e.g., 24 months), estimates by birth year might be slightly lower for some vaccines than were estimates by survey year, which on average, assessed vaccination by age 27.5 months. Trends in vaccination coverage by birth year and survey year are similar (*8*). Other changes include addition of assessment of ≥2 HepA doses by age 35 months to better reflect current ACIP recommendations and the addition of vaccination with 2 doses of influenza vaccine by age 24 months.***

The findings in this report are subject to at least two limitations. First, as with previous NIS-Child estimates by survey year, vaccination coverage estimates by birth year might be biased because of an incomplete sample frame, nonresponse, and underascertainment of vaccination (*6*). No evidence for change in survey accuracy from 2017 to 2018 was detected. Second, starting in 2018, the NIS-Child sample was drawn only from cellular telephone numbers. Vaccination coverage trends should thus be viewed with caution, although the effect of dropping the landline sample is likely small.

Improvements in childhood vaccination coverage will require that parents and other caregivers have access to vaccination providers and believe in the safety and effectiveness of vaccines. Increased opportunity for vaccination can be facilitated through expanded access to health insurance, greater promotion of available vaccines through the Vaccines for Children program, and solutions to logistical challenges such as transportation, child care, and time off from work. Providers can improve vaccination coverage overall and reduce disparities by administering all recommended vaccines during office visits. Compelling and accessible educational materials, combined with effective techniques for providers to use when discussing vaccination, can be used to counter inaccurate claims and communicate the value of vaccines in protecting the health of children (*9*). In addition, actionable data at a local level are needed so that interventions can be targeted to areas at risk for outbreaks of measles and other vaccine-preventable diseases. More immunization information systems will contribute to this effort because they streamline their data collection processes and improve data quality (*10*).^†††^ Given low survey response rates, CDC is working to better assess accuracy of NIS-Child vaccination coverage estimates, evaluate new survey approaches (e.g., switching to an address-based sample frame), and integrate data from immunization information systems and, potentially, other data sources (*7*).

**Summary**

**What is already known about this topic?**

The Advisory Committee on Immunization Practices recommends that children be vaccinated against 14 potentially serious illnesses before age 24 months.

**What is added by this report?**

Among children born in 2015 and 2016, coverage was high and stable for most vaccines. There were sociodemographic disparities in coverage, especially by health insurance status. The proportion of completely unvaccinated children remained small.

**What are the implications for public health practice?**

Coverage can be improved with increased access to providers and health insurance, administration of all recommended vaccines during office visits, and more effective patient education about vaccine safety and efficacy. Actionable local level data are a priority for creating targeted interventions to prevent outbreaks of measles and other vaccine-preventable diseases.

## Footnotes

* Estimates for states, selected local areas, and the territory of Guam are available online at https://www.cdc.gov/vaccines/imz-managers/coverage/childvaxview/data-reports/index.html. Certain local areas that receive federal Section 317 immunization funds are sampled separately and included in the NIS-Child sample every year (Chicago, Illinois; New York, New York; Philadelphia County, Pennsylvania; Bexar County, Texas; and Houston, Texas). Other local areas in Texas have been sampled in some survey years and not others, including El Paso County (survey years 2014–2017); Dallas County (survey years 2016 and 2017); Hildago County (survey years 2015 and 2018); Tarrant County (survey year 2018); and Travis County (survey year 2017). The NIS-Child was also conducted in Guam, Puerto Rico, and U.S. Virgin Islands; however, data collection in Puerto Rico and U.S. Virgin Islands was suspended during 2017 because of the severity of the hurricane season and did not occur at all in 2018, resulting in insufficient data for estimation of vaccination coverage by 24 months among children born during 2015–2016. National estimates in this report exclude all territories.

† NIS-Child used a landline-only sampling frame from 1995 through 2010. From 2011 through 2017, the survey was conducted using a dual-frame design, with both cellular and landline sampling frames included. In 2018, the NIS-Child returned to a single-frame design, with all interviews conducted by cellular telephone.

§ Children with at least one vaccination reported by a provider and those who had received no vaccinations were considered to have adequate provider data. “No vaccinations” indicates that the vaccination status is known because the parent indicated there were no vaccinations and the providers returned no immunization history forms or returned them indicating that no vaccinations had been given.

¶ The Council of American Survey Research Organizations (CASRO) household response rate is calculated as the product of the resolution rate (percentage of the total telephone numbers called that were classified as nonworking, nonresidential, or residential), screening completion rate (percentage of known households that were successfully screened for the presence of age-eligible children), and the interview completion rate (percentage of households with one or more age-eligible children that completed the household survey). The CASRO household response rate is equivalent to the American Association for Public Opinion Research type 3 response rate http://www.aapor.org/AAPOR_Main/media/publications/Standard-Definitions20169theditionfinal.pdf. For CASRO response rates and the proportions of children with household interviews that had adequate provider data for survey years 2013–2017, see: https://www.cdc.gov/vaccines/imz-managers/nis/downloads/NIS-PUF17-DUG.pdf, (Appendix G).

** https://www.cdc.gov/vaccines/imz-managers/coverage/childvaxview/pubs-presentations/NIS-child-vac-coverage-estimates-2014-2018.html.

†† The combined 7-vaccine series (4:3:1:3*:3:1:4) includes ≥4 doses of diphtheria and tetanus toxoids and acellular pertussis vaccine; ≥3 doses of poliovirus vaccine; ≥1 dose of measles-containing vaccine; ≥3 or ≥4 doses (depending upon product type) of *Haemophilus influenzae* type b conjugate vaccine; ≥3 doses of hepatitis B vaccine; ≥1 dose of varicella vaccine; and ≥4 doses of pneumococcal conjugate vaccine.

§§ MSA status was determined based on household reported city and county of residence and was grouped into three categories: MSA principal city, MSA nonprincipal city, and non-MSA. MSAs and principal cities were as defined by the U.S. Census Bureau (https://www.census.gov/programs-surveys/metro-micro.html). Non-MSA areas include urban populations not located within an MSA as well as completely rural areas.

¶¶
https://www.cdc.gov/vaccines/programs/vfc/index.html .

*** This measure of influenza vaccination differs from other estimates from NIS-Flu (see https://www.cdc.gov/flu/fluvaxview/coverage-1718estimates-children.htm): it is based on provider-reported vaccinations instead of relying on parental report; and it reflects vaccinations that might have been received over two influenza seasons, while NIS-Flu estimates are for one season. Receipt of two influenza vaccinations by age 24 months is also a Healthcare Effectiveness Data and Information Set measure (https://www.ncqa.org/hedis/measures/childhood-immunization-status/); this measure can be used to identify commercial and Medicaid health plans within states with lower vaccination coverage.

††† General information about immunization information systems is available at https://www.cdc.gov/vaccines/programs/iis/about.html. Guidance on using immunization information systems to identify geographic areas of populations at risk for outbreaks of vaccine-preventable diseases is available at https://repository.immregistries.org/files/resources/5bae51a16a09c/identifying_immunization_pockets_of_need-_final2.pdf.
